# Trust in the smart home: Findings from a nationally representative survey in the UK

**DOI:** 10.1371/journal.pone.0231615

**Published:** 2020-05-29

**Authors:** Sara Cannizzaro, Rob Procter, Sinong Ma, Carsten Maple

**Affiliations:** 1 Warwick Manufacturing Group, University of Warwick, Coventry, England, United Kingdom; 2 Computer Science, University of Warwick, Coventry, England, United Kingdom; 3 Computer Science, University of Warwick, Coventry, England, United Kingdom; 4 Queen's Management School, Queen’s University Belfast, Belfast, Northern Ireland, United Kingdom; 5 Warwick Manufacturing Group, University of Warwick, Coventry, England, United Kingdom; University of Milan, ITALY

## Abstract

Businesses in the smart home sector are actively promoting the benefits of smart home technologies for consumers, such as convenience, economy and home security. To better understand meanings of and trust in the smart home, we carried out a nationally representative survey of UK consumers designed to measure adoption and acceptability, focusing on awareness, ownership, experience, trust, satisfaction and intention to use. We analysed the results using theories of meanings and acceptability of technologies including semiotics, social construction of technology (SCOT) and sociotechnical affordance. Our findings suggest that the meaning and value proposition of the smart home have not yet achieved closure for consumers, but is already foregrounding risks to privacy and security amongst the other meaning-making possibilities it could afford. Anxiety about the likelihood of a security incident emerges as a prominent factor influencing adoption of smart home technology. This factor negatively impacts adoption. These findings underline how businesses and policymakers will need to work together to act on the sociotechnical affordances of smart home technology in order to increase consumers’ trust. This intervention is necessary if barriers to adoption and acceptability of the smart home are to be addressed now and in the future.

## 1. Introduction

The Internet of things (IoT) refers to the many uses and processes that result from giving a network address to a digital device and equipping it with sensors [1]. Businesses and government expect it to be an increasingly important driver of product and service innovation–and hence economic growth–in the next few years. Understanding what factors might shape IoT adoption is very important if barriers are to be addressed and incentives refined. Managing innovation is challenging [2] and, as IoT is a convergence of ideas and technologies developed from a number of domains [3], its adoption and acceptability may be particularly complex. In this article, we report on study of IoT adoption in relation to the smart home, which was conducted as part of the research programme of PETRAS, the UK’s Research Hub for IoT.

Using a nationally representative survey of UK consumers, our study aims to identify factors that influence meanings of and trust in the smart home. By so doing, we seek to overcome shortcomings of previous IoT adoption studies that have simply conflated ‘adoption’ with ‘acceptance’, since the latter notion does not sufficiently *problematize* [4] the conceptual and political values that it takes for granted [5]. Drawing on theories of meanings, ethics and acceptability of technologies, including the Social Construction of Technology (SCOT) [6] we aim to explore the how consumers make sense and trust the smart home. We discuss the implications of our findings for businesses and government innovation policy. Finally, we outline how our approach helps to identify social divides that are likely to characterise the UK population and impact on IoT adoption in the years to come.

## 2. Literature review

### 2.1 Why adoption is not enough (the problem with adoption studies)

The ‘smart home’ can be defined as the integration of Internet-enabled, digital devices with sensors and machine learning in the home [7]. The aim of smart home devices, as conceived by industry, is to provide enhanced entertainment services, easier management of domestic chores and protection from domestic risks, including flood, burglary and fire, and to lower energy consumption. The smart home is a key application domain for IoT (the others being industry, cities, health and environment) and includes a wide range of devices, such as smart speakers and hubs, lighting, sensors, door locks and domestic appliances. In 2018, smart speakers and hubs were present in 15.8 million homes in Europe and the global market for this segment of smart home technology is forecast to treble in size by 2023 [8]. However, despite statements of value by ‘powerful stakeholders’, such as business and governmental organisations, the meaning and value for the consumer, including trust, remains uncertain.

Attempts have been made in the adoption literature to map such meanings, values and trust. This perspective on adoption is typically associated with studies of how technological innovations diffuse through and are taken up within society. It is defined as a process “starting with the user becoming aware of the technology, and ending with the user embracing the technology and making full use of it” [9]. This process has traditionally been visualised as a normal, bell-shaped curve, initially rising slowly when there are very few adopters (‘innovators’ and ‘early adopters’), then accelerating to a peak as the ‘early majority’ join and, thereafter increasing slowly with the addition of the ‘late majority’ and ‘laggards’ [10]. In order to understand where the adoption of smart home IoT stands on the curve, the first question addressed by this study is: *to what extent has the smart home been adopted in the UK*?

Several recent studies have been carried out specifically on adoption of IoT [7, 11, 12, 13, 14, 15, 16, 17, 18, 19]. Some of these treat acceptance and adoption as synonyms [e.g., 7, 12, 14]. Theoretically, this approach is a shortcut: when adoption and acceptance are conflated, definitions of adoption quickly turn to *factors* for adoption or for technology acceptance (for example, see [20]). However, despite its methodological sophistication, this approach emphasises quantitative testing of acceptance, omitting any references to theories of meanings of technology.

Furthermore, adoption studies are typically carried out by what Rogers [10] has called ‘change agencies’, whose short-term goal is to facilitate clients’ adoption of innovations. Such agencies often follow a segmentation strategy of least resistance for the innovations they aim to establish[10]. This means that short-term economic gain is the likely rationale for changes effected through adoption. This logic also appears in recent key IoT adoption studies [e.g., 16, 17, 7], which justify adoption purely through economic arguments. Arguably, it is because of this purely economic framing that adoption of IoT has not so far been grounded in a solid theoretical tradition.

This narrow vision ignores the long-term well-being of people and their environment. Therefore, IoT adoption is at risk of becoming a ‘technocratic management strategy’ [21] that ignores consumers’ values and feelings–in other words–their role in constructing and contributing to the meanings of the technology, and hence its further design and development. This technocratic perspective does not sufficiently problematize the technology, with serious implications for its future impact on society [22, 23, 24, 25]. We therefore set out to embed empirical investigation within a few ideas concerning theories of meanings in technology.

### 2.2. Theories of meanings and acceptability of technologies

To start to understand meanings in technology, one ought to consider the ‘meaning of meaning’ in general. This is a large and intensive quest in areas such as semiotics and linguistics, and this paper is not the place to provide an extensive review of the concept; however, for our present purpose, it suffices to say that studying meaning(s) requires at the very least an understanding of a) the nature of interpretation and b) factors that constrain this phenomenon. As semiotician Eco [26] put it, the process of interpretation does not consist of an unlimited drift of meaning, a process where anything can mean anything; rather, it is constrained by limits. In anthropology, the nature of such limits was initially called ‘context’ [27]. Contextual factors constraining yet at the same time, enabling the emergence of meaning, are diverse, and include feelings, ecological and physiological constraints, interpretational errors and ‘observership’ [3]. Simply put, meaning is a relational phenomenon emerging out of the interplay of these constraints. In a technological context, one could argue that meaning emerges across the whole socio-technical-ecological system, including users (their psychology and physiology), the technology, their environment and their unique perspective in the environment (observership). For the sake of simplicity, in this study we will refer to this socio-technical-ecological system as ‘meanings in the smart home’. Within innovation studies, approaches to understandings meanings range from technological determinist [e.g., 28] to social constructivist [e.g., 29]. Occupying a conceptual middle ground are the Social Construction of Technology (SCOT) framework [6] and ideas in affordance theory [30]. In SCOT, a key concept is ‘interpretive flexibility’ [31], which recognises that the ‘meaning’ of an innovation maybe initially be contested by different stakeholders before ‘closure’–and hence its use value–is reached [32]. SCOT originated in studies of organisational innovation processes and some argue that its translation to the consumer digital technology marketplace requires a new framework variant: Social Construction of Digital Technologies (SCODT). The SCODT framework is based on four dimensions: technologies (digital ecosystems), interactions (interpersonal, person–technology, technology–technology, technology–physical environment), social groups (networked individualism bypassing established communication hierarchies) and context (socio-digital environment) [2]. Our research is located along the dimension of person–technology interactions, as it seeks to map out the meaning that consumers confer on the smart home.

Affordance also investigates meanings in technologies by positing that the materiality of technology influences, but does not determine, the interpretive possibilities for users [33]. Originally, Gibson [30] defined affordance as an action possibility in the environment, Norman [34] defined it as the design aspect of an object, Gaver [35] and Leonardi [36] as a relational phenomenon emerging through direct interaction with the technologies. Bradner [37] expanded the concept from ‘object affordance’ to ‘social affordance’ in order to capture the social dimensions of technology use. More recently, Vatrapu [38] has broadened it still further to emphasise experience formation in and through interaction with technologies, defining sociotechnical affordances as action-taking possibilities and meaning-making opportunities in a socio-technical system relative to actor competencies and system capabilities. This is a useful perspective because identifying social affordances allows building a generalizable description of broader sociotechnical factors on adoption that includes meanings of and trust in technology, as we set out to do in this study. Specifically, this allows us to identify which factors become more salient for consumers, and hence a likely point of action in regard with adoption or non-adoption.

In this study, meanings in the smart home are examined in light of the interpretational constraints referred to in the technology literature as *acceptability factors*. This concept adds a specific *ethical* context to the adoption process [39] since acceptability can be defined as a judgement that prescribes how the technology examined ought to be desirable, either instrumentally or morally. Acceptability is a popular perspective in assistive technology studies [e.g., 40, 41, 15].

Owing to tension between ethical and strictly commercial views in IT innovation [42] ethical considerations remain somewhat scarce in IoT studies (exceptions being [18], [15]). Existing acceptability studies of the smart home, particularly those focusing on areas of acceptability such as trust, are limited to non-academic sources [43]. Although some of these are substantial–for example, a study by TechUK [44] was based on a sample of more than 1000 British consumers–they are not theoretically grounded and their statistical significance is unclear.

Furthermore, as a particular instance of IoT, the smart home presents unique challenges and therefore requires careful consideration of consumers’ interpretations. That is because, in a business transaction involving consumers and IoT manufacturers/service providers, the product is no longer the technology itself, but instead it is the consumer, a phenomenon sometimes labelled as people-based marketing [45]. Furthermore, unlike previous digital technologies, data are generated through not only consumers’ intentional use of smart devices, but also their unintentional use. As sensors are continuously monitoring consumers’ behaviour, data may be generated unconsciously, transmitted and processed without their full awareness. Trust in the security, privacy and even reliability of the devices, and interpretation of the risks that the technology affords, therefore, uniquely frame the acceptability of the IoT.

This article presents a novel *joint adoption and acceptability study* where we seek to map out factors at play in the acceptability of the smart home as viewed in relation to consumers’ meanings and trust.

## 3. Methodology: Sampling and questionnaire theoretical constructs

### 3.1 Sampling

In order to contextualise IoT adoption from a user-centred acceptability perspective, we devised a survey investigating consumers’ responses and attitudes to smart home devices. This study involving Human Subject Research received full approval by the University of Warwick ethics committee on 17 July 2018 (ref. no REGO-2018-2219). To develop our sampling strategy, existing IoT empirical studies were reviewed and summarised. A systematic review of the first 50 results of the top ten information systems (IS) and management journals in 2017 and 2018 (according to Scimago Journal and Country Rank) produced only three empirical studies of IoT adoption [16, 17, 7], two of which are quantitative, involving surveys, and one qualitative, involving interviews. The samples used in the surveys had 498 [17] and 269 respondents [7] respectively, suggesting a gap in the research literature for studies with a nationally representative sample.

We also reviewed sources in other journals and included less recent empirical studies. The sample sizes for these surveys were 204 [46], 321 [47], 346 [48], 368 [49], to 426 respondents [50]. This additional search confirmed the gap in studies of a scale that might be considered as a nationally representative sample.

We interrogated the survey results based on demographic factors (gender, age and education) that have previously been found to affect the relationship between people and technology use. Previous research has found that “gender plays an important role in shaping individual technology adoption” [51], that it is more difficult for older people to adopt new technologies [52], and that education influences adaptation to change, including changes brought about by new technologies [53].

A nationally representative sample based on these demographic factors was recruited through the online market research sample aggregator Qualtrics. This service builds samples from more than 20 online panels to ensure the most diverse, representative data sets, through adverts on social media, google search, and on websites. While we cannot fully control for the population that may be excluded (e.g. the Internet or computer illiterate), we are still confident for the sample to be representative since 95% of adults aged 16 to 74 years (the demographics captured by our survey) in the UK in 2018 were internet users—hence they could have had the opportunity to be exposed to the panel recruitment adverts [54]. In total, 2,101 participants were recruited, based on representative quotas. Given the large sample, the extra 68 participants recruited on top of the representative sample (n = 2033, see Table 1) were not found to affect the representative quotas in any way. The quotas were based on 2016 European statistical datasets for population by age and gender, and by age sex and educational attainment level. Note that ISCED 0–2 includes Pre-primary education, Primary Education and GCSE/Vocational GCSE or equivalent (incl. O-levels). ISCED 3–4 includes A-level/Vocational A-level or equivalent (incl. AS-level), Higher Diplomas below degree level/as gateways to degree. ISCED 5–6 includes Undergraduate degree and Postgraduate degree (Master and PhD).

**Table 1:** Summary of survey respondents by gender, age and education.

**Gender**		
Male	992	49%
Female	1041	51%
**Total**	**2033**	
**Age**		
18–24	228	11%
25–34	341	17%
35–49	516	25%
50–64	482	24%
65+	466	23%
**Total**	**2033**	
**Education**		
ISCED 0–2	429	21%
ISCED 3–4	820	40%
ISCED 5–6	784	39%
**Total**	**2033**	

### 3.2 Questionnaire design: Theoretical constructs

To explore the smart home as a socially constructed artefact, and to map the meaning that people assign to the smart home, we identified and focused on some key contextual factors suggested by the literature on technology adoption and acceptability. Based on these contextual factors, we created a questionnaire in English with 17 questions. The specific survey items we asked, and their corresponding variables in our analysis, are presented in Table 2. Three of these questions were reserved solely for people with experience of using smart home devices. The questionnaire was internally reviewed for content validity, pilot tested internally and then soft launched to an initial group of 52 consumers. Items for the questionnaires were based on various theoretical perspectives on adoption and acceptability, as outlined below.

**Table 2:** Summary of variables.

Variable Name	Corresponding survey items
*Awareness (A)*	
Awareness IoT	A1 Have you heard of the expression ‘Internet of things’
Awareness smart home	A2 Have you heard of the expression ‘smart home?’
Ownership (O)	
Ownership experience	03 The following are examples of Internet of things/smart home devices. Please indicate whether you own and use the specified device. (note: if you have experience of using someone else’s smart home device but you do not own one yourself, select either ‘do not own’ or ‘previously owned’, or ‘undecided’).
Ownership amount	04 How many smart devices from the above list do you currently own, including any duplicate devices? (for example, if you own two smart TVs and one Google Home personal assistants, put ‘3’ in the box)
*Experience of Use I*	
Experience length	E5 How long have you been using smart home devices in your own home?
Experience routine	E6 The use of the smart features of my smart home devices has become routine
Experience functionalities	E7 How many of the functionalities of your smart home device do you use?
*Trust in general (T)*	
Trust in competence	T8 I (would) fully trust smart home devices not to fail, and to function as I expect them to–(competence)
Trust in benevolence	T9 Knowing that smart home devices allow companies or organisations to collect data about how I use them, and hence about my domestic habits, would restrict me from owning/using them (benevolence)
Trust in integrity	T10 I would trust companies not to use data produced by smart home devices for any purpose without my explicit consent (integrity)
*Trust in Privacy (T)*	
Likelihood of privacy breach	T11 I think the likelihood of the security of smart home devices being compromised and resulting in a privacy/data breach is high
Impact of privacy breach	T12 I think the impact of the security of smart home devices being compromised and resulting in a privacy/data breach is low
Impact of controversy	T13 The Facebook user data sharing controversy makes me less willing to own/use smart home devices
*Trust in security (T)*	
Likelihood of incident	T14 I think the likelihood of the security of smart home devices being compromised and resulting in an incident (e.g. burglary, fraud) is high
Impact of incident	T15 I think the impact of the security of smart home devices being compromised and resulting in an incident (e.g. burglary, fraud) is low
*Satisfaction (S)*	
Satisfaction	S16 I find that my smart home devices exceeded my expectation
*Future intention to use and recommendation*	
Intention to use	I17 I intend to use or continue using smart home devices in the future
Intention to recommend	I18 I would not recommend smart home devices to my friends

#### 3.2.1 Awareness

Awareness has been seen as critical to developing new ICT infrastructures [55] and as a key determinant of consumers’ adoption behaviour [56]. Lack of awareness has been identified as an obstacle to mobile phone adoption [57]. To measure awareness of smart home devices, we focused on terms in common use, such as ‘Internet of things’ and ‘smart home’.

#### 3.2.2 Ownership

We measured ownership in a broadly similar way to that used by Venkatesh et al. [58] to measure consumer technology usage. Hence, we combined variety (breadth) of devices owned with whether respondents had owned them in the past, and if they did not, whether they wished to do so (depth).

#### 3.2.3 Experience of use (early adoption, smart functionality usage, habit)

Experience of use has been defined as the passage of chronological time from initial use of a technology [58]. Based on this, we asked respondents how long they had been using smart home devices. We used this measure to identify innovators, early adopters, early and late majority and laggards [10]. To identify innovators and early adopters using the results for the ‘ownership’ (breadth) variable in our survey, we looked into the history of the release onto the UK market of the three most popular types of smart home device: smart TVs, smart meter and smart assistant (specifically Amazon Echo). These dates were 2012 [59], 2013 [60], and September 2016 respectively [61]. We identified the latter as the point when the idea of the smart or connected home entered public consciousness, first because Amazon Echo is designed to connect to several devices rather than just the Internet router and, second because, since Amazon Echo is the third most popular device connected to the Internet, it marks the point at which the smart home became a reality. Hence, we considered people owning IoT devices in or before 2016, that is having more than two years of smart home experience, to be innovators and early adopters.

To identify the early majority, i.e. those who follow with deliberate willingness but seldom lead in adopting innovations [10], we looked at respondents who had adopted smart home devices during the year before data collection took place (2018).

Finally, we identified those who had no experience of smart home devices as the late majority or laggards. The late majority usually adopts new technology when the weight of ‘system norms’ favour the innovation [10]. In the UK, the system norm may be considered to be the government’s *Code of Practice for Consumer IoT security* [24], which was first published in March 2018, just a few months before data collection took place. Hence, we considered people who have not adopted before this norm was published, to constitute the late majority or laggards.

We also measured functionality usage [58] by asking respondents to indicate how many of the functions of their smart home devices they used. We measured the extent to which respondents felt that their use of their devices’ smart features had become routine, since habit necessarily relates to experience [62] and has a direct effect on technology use [58].

#### 3.2.4 Trust components

Trust is fundamental to consumer technology where transmission of personal and sensitive information is involved. It is a key aspect of the acceptability of IoT, as a technology must be trustworthy for it to be desirable or acceptable. Trust refers to the belief that an entity will act cooperatively to fulfil clients’ expectations without exploiting their vulnerabilities [63].Trust can be broken down into *competence* (e.g., belief in the capability to protect clients’ personal and sensitive data), *benevolence* (belief that clients’ interests will be respected) and *integrity* (belief that the entity is honest and will fulfil its promises to the client) [64]. Our survey mapped levels of trust across these three basic components. With regard to competence, we focused on ability to perform automated functions reliably, a measure of ‘trust in automation’ [65]. Hence, we measured attitudes toward automation that affect reliance [66]. With respect to benevolence and integrity, we focussed on privacy.

Privacy and security are expected to be key factors affecting acceptance, and hence adoption, of the smart home [12]. To date, there have been few non-technical studies of the security and privacy concerns of smart home device users [67]. In regard with privacy, testament to the importance of trust in the privacy-preserving capability of devices is the fact that the year in which we designed this study is the year in which GDPR came into existence, an event which made ‘privacy by design’ a legal requirement across Europe. Security is among the biggest obstacles standing in the way of full adoption of IoT [68]. Misra et al. [12] state that threats to IoT are more severe than to those of other technologies, as their realization may also impact on the physical world. For example, if a smart home security is compromised, it may be possible to control smart door locks. All trust-related questions were considered alongside perceived risk, because perceived risk is an important component of technology acceptance and adoption and impacts on trust [69]. To qualify the type of risk incurred when using smart home devices, we referred to the measure of overall perceived risk in financial, performance, physical, psychological, social risk as in [70].

#### 3.2.5 Satisfaction

Satisfaction is linked with the intention to continue using the technology [71] Expectation and Confirmation Theory (ECT) [72] posits that consumers form a satisfaction assessment, which, in turn, affects their repurchasing intentions. We measured levels of satisfaction in relation to intention to use the technology, as well as to levels of trust [71, 73, 74,75].

#### 3.2.6 Intention to use in the future and intention to recommend to others

Venkatesh et al. [20] underline the importance of the role of intention to use or continue using the technology as a predictor of usage, which may be linked to adoption. A related yet different take on adoption from the perspective of technology rejection theory, is the non-acceptance rate [76, 77, 78]. Social learning, where individuals learn from the experiences of others, including negative experiences was expected to affect adoption (35; 18]. Hence, we measured technology resistance in terms of whether respondents would recommend smart home devices to friends.

## 4. Results and analysis

### 4.1. Descriptive statistics results

We used the Chi-squared to test whether gender, age or education were statistically significant factors in participants’ responses. The results for questions based on a small number of ordinal values, such as Yes/No questions, are presented as percentages, whereas results for questions based on a Likert scale are presented as mean and standard deviation. Table 3 illustrates the descriptive statistics of our variables.

**Table 3:** Descriptive statistics.

Variable	Overall	Gender		Age			Education		
		Female	Male	18–24	35–49	65+	ISCED 0–2	ISCED 3–4	ISCED 5–6
O4	1.86	1.86	1.87	2.59	1.86	1.39	1.45	1.95	2.01
E6	2.89	2.89	2.88	2.71	2.72	3.22	2.97	2.92	2.82
	(1.19)	(1.18)	(1.19)	(1.17)	*(1*.*15)*	*(1*.*22)*	(1.17)	(1.22)	(1.16)
E7	3.03	3.06	3.00	2.85	3.04	3.15	3.16	3.04	2.96
	(1.09)	(1.10)	(1.08)	(1.07)	(1.06)	(1.17)	(1.22)	(1.10)	(1.01)
T8	3.37	3.39	3.34	3.16	3.35	3.54	3.32	3.40	3.36
	(1.15)	(1.13)	(1.17)	(1.20)	(1.15)	(1.09)	(1.10)	(1.15)	(1.17)
T9	2.37	2.37	2.37	2.55	2.38	2.25	2.44	2.35	2.35
	(1.07)	(1.03)	(1.11)	(1.09)	(1.06)	(1.06)	(1.05)	(1.05)	(1.10)
T10	3.22	3.16	3.28	2.84	3.17	3.40	3.09	3.25	3.26
	(1.28)	(1.27)	(1.29)	(1.33)	(1.27)	(1.24)	(1.25)	(1.26)	(1.32)
T11	2.29	2.26	2.31	2.32	2.28	2.25	2.31	2.31	2.25
	(0.95)	(0.91)	(0.99)	(0.93)	(0.97)	(0.94)	(0.96)	(0.93)	(0.96)
T12	3.33	3.35	3.32	3.26	3.35	3.43	3.39	3.31	3.33
	(1.09)	(1.05)	(1.12)	(1.06)	(1.09)	(1.06)	(1.04)	(1.10)	(1.10)
T13	2.44	2.47	2.41	2.47	2.55	2.26	2.48	2.45	2.42
	(1.09)	(1.07)	(1.10)	(1.08)	(1.09)	(1.07)	(1.10)	(1.05)	(1.11)
T14	2.50	2.48	2.52	2.50	2.52	2.48	2.48	2.50	2.50
	(1.02)	(1.00)	(1.04)	(1.01)	(1.02)	(1.02)	(0.98)	(1.03)	(1.03)
T15	3.32	3.32	3.32	3.26	3.34	3.41	3.31	3.28	3.36
	(1.10)	(1.08)	(1.12)	(1.14)	(1.11)	(1.08)	(1.06)	(1.13)	(1.08)
S16	2.88	2.83	2.94	2.47	2.75	3.20	2.86	2.91	2.85
	(1.02)	(0.98)	(1.05)	(0.98)	(0.97)	(1.03)	(0.99)	(1.04)	(1.00)
I17	2.45	2.44	2.46	1.97	2.41	2.84	2.68	2.41	2.37
	(1.21)	(1.21)	(1.21)	(1.01)	(1.20)	(1.23)	(1.22)	(1.20)	(1.19)
I18	3.03	3.10	2.95	3.33	3.09	2.74	2.93	3.02	3.09
	(1.18)	(1.11)	(1.24)	(1.16)	(1.17)	(1.19)	(1.14)	(1.16)	(1.21)

Means and (standard deviations) are shown for variables. ISCED levels as defined in footnote 4 refer to low (0–2), medium (3–4) and high (5–6) level of education. Results in grey-shaded areas are significant at p < 0.01.

#### 4.1.1 Awareness

**Fig 1:**
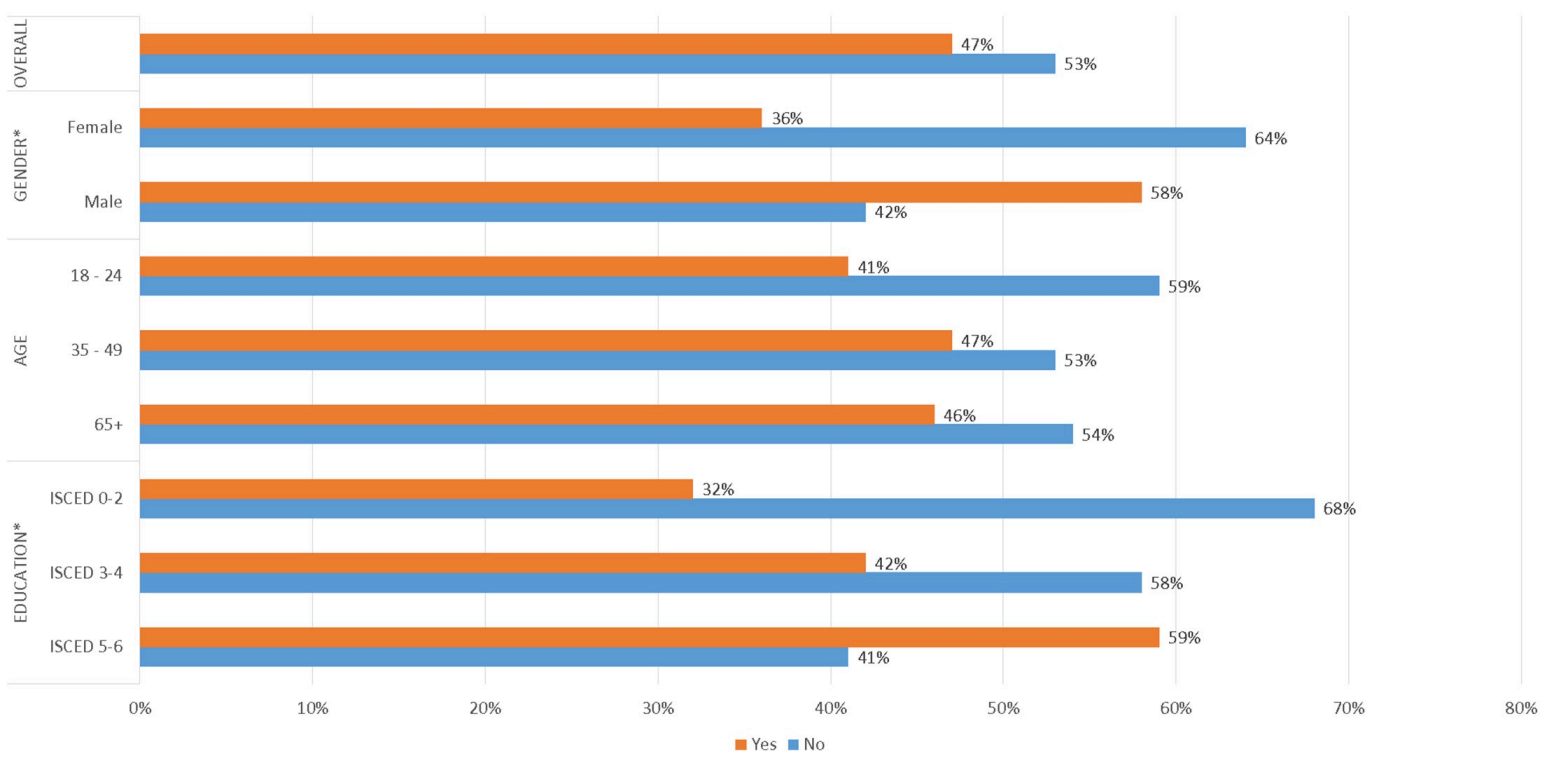
Results for awareness of the expression ‘Internet of things’ (Q1).

Fig 1 (A1) shows that more male (58%) than female respondents (36%) were aware of the expression ‘Internet of things’. These differences are significant at p < 0.01. More 35–49 year-old respondents (47%) and respondents who were 65 or older (46%) were aware of the expression ‘Internet of things’ than 18–24 year-old respondents (41%). However, these differences are not statistically significant. There are more ISCED 5–6 respondents (59%) who are aware of the expression ‘Internet of things’ than ISCED 3–4 (42%) and ISCED 0–2 respondents (32%). These differences are significant at one per cent level.

**Fig 2:**
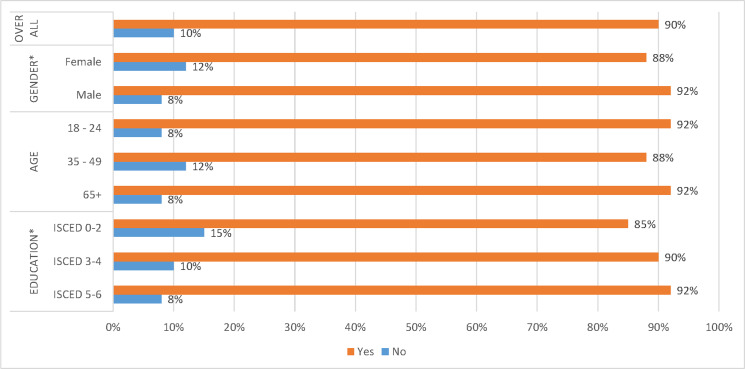
Results for awareness of the expression ‘smart home’ (Q2).

Fig 2 (A2) shows that slightly more female (12%) than male respondents (8%) were unaware of the expression ‘smart home’. These differences are significant at the one per cent level. Slightly more 35–49 year old respondents (12%) have not heard of the expression ‘smart home’ than 18–24 year old respondents (8%) and respondents 65 or older (8%). However, these differences are not statistically significant. More ISCED 0–2 respondents (15%) were unaware of the expression ‘smart home’ than ISCED 3–4 respondents (10%) and ISCED 5–6 respondents (8%). These differences are significant at p < 0.01.

#### 4.1.2 Ownership

**Fig 3:**
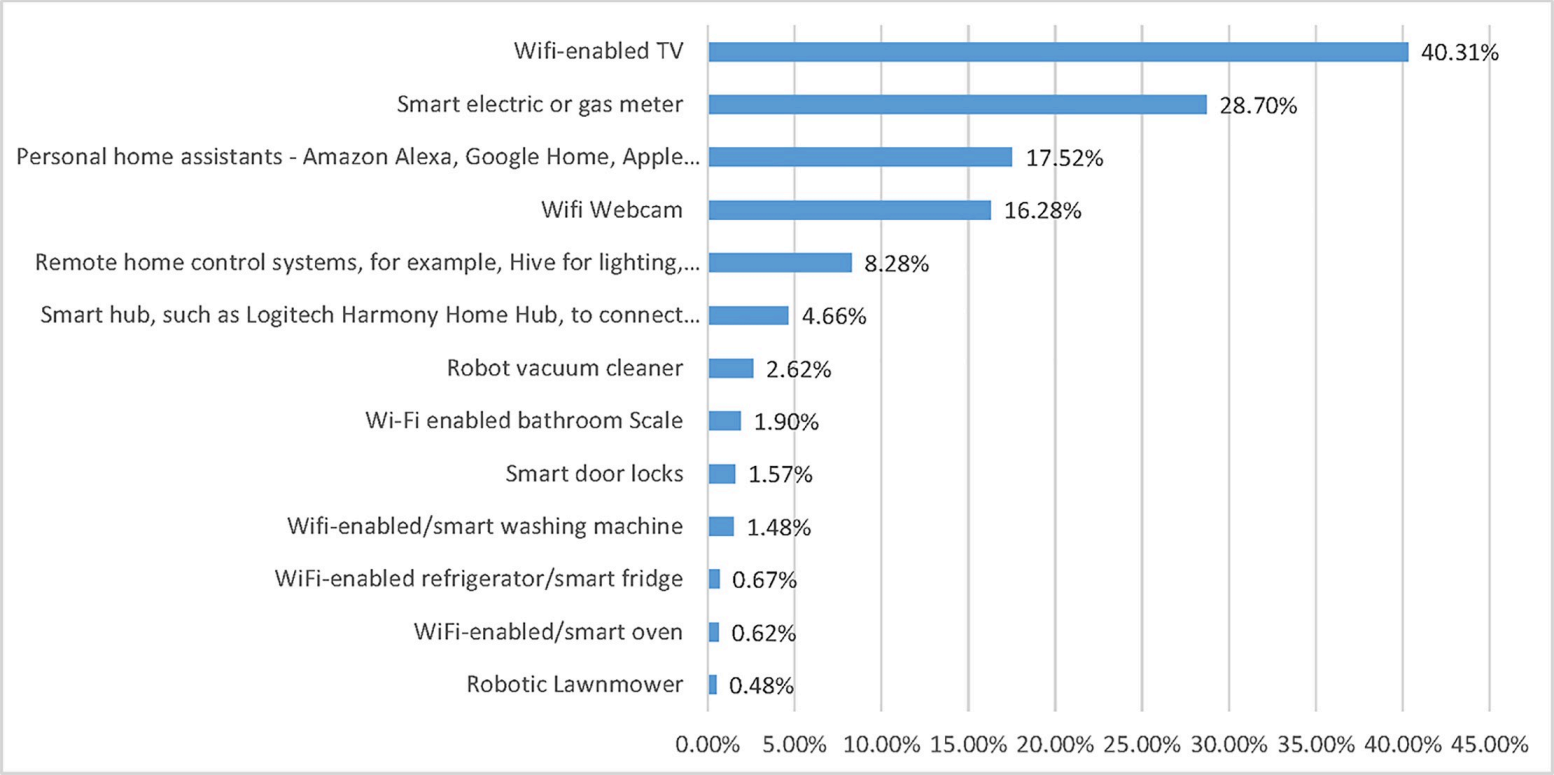
Overall results for ownership and usage of specific smart home devices (03).

In Question 3 (03) respondents were asked to select from a list of 13 smart home devices the ones they currently used (Fig 3). The most owned and used smart home device was the smart TV (40%), followed by the smart meter (29%) and the personal home assistant (16%). The rankings are consistent across age demographics, with the exception of respondents 65 or older. In fact, the third most-used devices amongst respondents aged 65 or older was the wireless webcam (19%) rather than the personal home assistant.

Results for 04 (Table 3) shows that, on average, respondents owned 1.86 smart home devices. This is unaffected by gender but is affected by age: respondents aged 18–24 tended to own above average (2.59), while respondents aged 65 plus tended to own fewer than the average (1.39). The overall mean of device ownership also varies with educational level, with respondents educated to ISCED 3–4 or ISCED 5–6 level owning more devices (1.95 and 2.01 respectively) than those educated to ISCED 0–2 level (1.45).

#### 4.1.3 Experience of use

**Fig 4:**
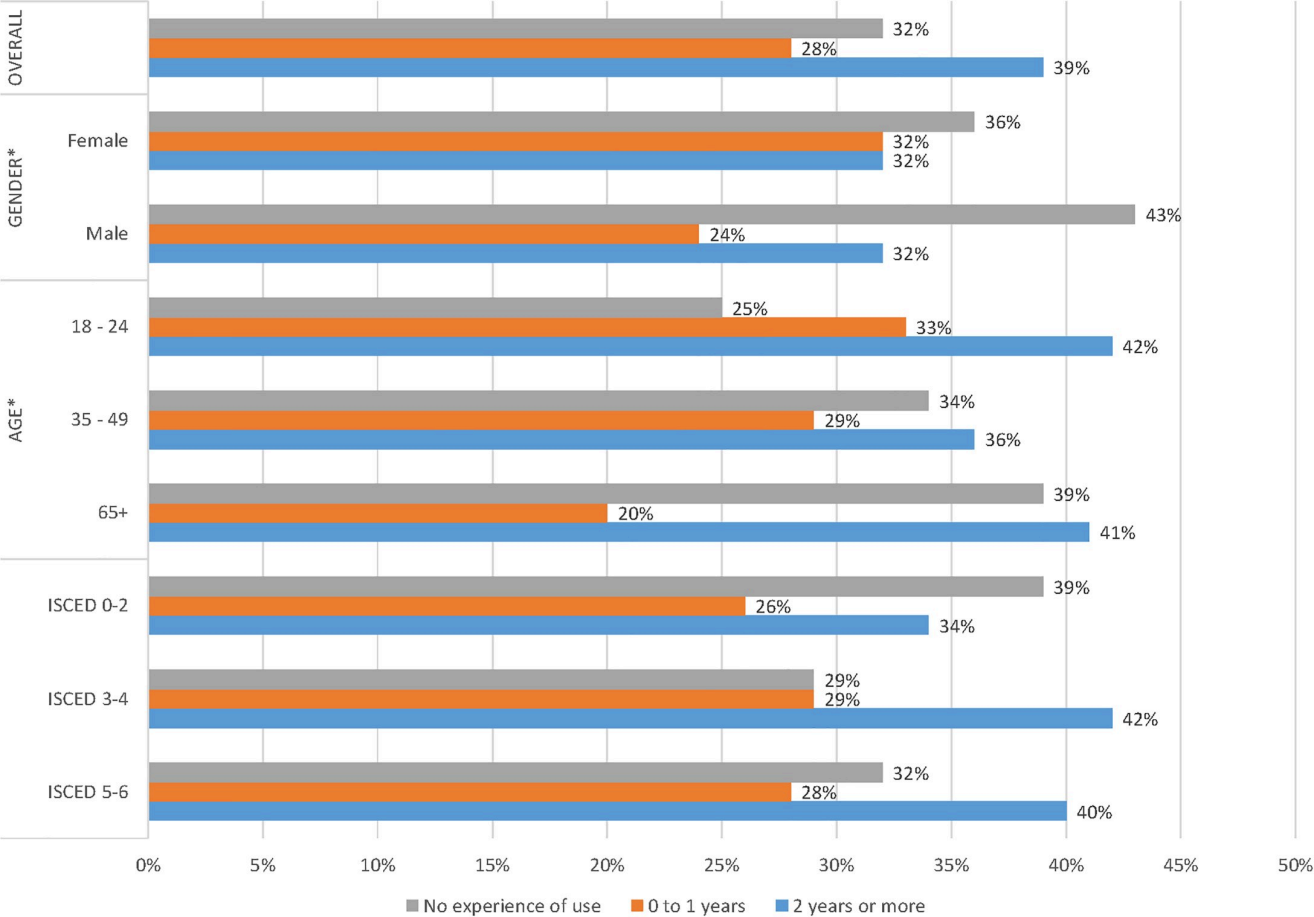
Experience of use indicating both the amount of experienced versus inexperienced smart home users and the time in which the technology was first adopted. (Q = E5).

Experience of use measured the number of experienced versus the number of inexperienced smart home device users, and when they were initially adopted. In total, 68 per cent of respondents reported that they use smart home devices. Fig 4 shows that, overall, 39 per cent of respondents have owned smart home devices for two or more years, 32 per cent did not own one yet, and 28 per cent had bought one in the past year.

Forty-three per cent of male respondents did not own smart home devices, compared with 36 per cent of female respondents. However, more female (32%) than male respondents (24%) reported having bought a device in the past year. Finally, equal proportions of female and male respondents (32%) had bought devices two or more years ago.

With regard to age, a lower percentage of respondents aged 18–24 (25%) had no experience of smart home devices than those aged 35–49 (34%) or 65 or older (39%). A higher percentage of respondents aged 18–24 (33%) or 35–49 (29%) had bought smart home device in the past year than respondents aged 65+ (20%). Finally, a higher percentage of respondents aged 18–24 (42%) or 65 plus (41%) have bought devices two or more years ago than respondents aged 35–49 (36%).

For education, a higher percentage of ISCED 0–2 respondents (39%) had no experience of use than respondents with ISCED 3–4 or ISCED 5–6 education (29% and 32%). The percentages of people who had bought a smart home device in the past year are broadly similar across education groups, ranging from 26%, 29% and 28% for ISCED 0–2, ISCED 3–4 and ISCED 5–6 respectively. ISCED 3–4 and ISCED 5–6 respondents (42% and 40% respectively) tend to have 2 or more years’ experience than those with ISCED 0–2 (34%). The results for gender, age and education are all statistically significant at p < 0.01.

Results for E6 (Table 3) shows that, overall, respondents with experience of smart home devices tend to mildly agree that their use had become routine (mean 2.89). This result is consistent for all independent variables (gender, age and education), except for those aged 65 plus, who felt more neutral or slightly disagreed that their device use had become into a stable habit (mean 3.22). The results for age and education are all statistically significant at p < 0.01.

Results for E7 (Table 3) show that, overall, users with experience of smart home devices tended to agree (mean 3.03) that they use only some of the functionalities. This result was consistent across gender (mean 3.06 for females and 3.00 for males), ISCED 3–4 respondents (3.04) and respondents aged 35–49 (mean 3.04). ISCED 0–2 respondents (mean 3.16) or 65 or over (3.15) tend towards using only a few functionalities. On the other hand, respondents aged 18–24 (2.85), as well as ISCED 5–6 respondents (2.96), tended to use most functionalities. The results for age are statistically significant at p < 0.01.

#### 4.1.4. Trust (General: Competence, benevolence, integrity)

We measured levels of trust in smart home devices according to its component elements of competence, benevolence, and integrity (T8, T9, T10). Question 8 (T8) elicited the average level of trust in devices performing automated functions reliably. Overall, respondents’ trust for this measure tended to be neutral to fairly low (mean 3.37). Respondents aged 65 and over tended to distrust more (mean 3.54) than other younger respondents, and ISCED 0–2 respondents distrusted less (mean 3.32) than others. The results for age and education are statistically significant at p < 0.01.

Question 9 (T9) measured the average level of trust in the benevolence of smart home devices, i.e. respondents’ belief that their interests would be respected. Overall, respondents’ trust as reflected in this measure was fairly low (mean = 2.37) across gender, for respondents aged 35–49 and for respondents with ISCED 3–4 or ISCED 5–6 education, and even lower for respondents aged 65 plus (mean 2.25). Respondents aged 18–24 and with ISCED 0–2 education tend to be slightly less distrusting (mean 2.55 and 2.44 respectively). The results for age and education are statistically significant at the one per cent level.

Question 10 (T10) measured the level of trust in the integrity of smart home devices. Overall, respondents’ trust as reflected in this measure tends to be fairly low (mean 3.22) and even lower for those aged 65 and over (mean 3.40). This result is broadly consistent across gender, respondents aged 35–49, as well as respondents with ISCED 3–4 and ISCED 5–6 education. As for benevolence, levels of trust in integrity tend to be slightly higher among respondents aged 18–24 (mean 2.84) and ISCED 0–2 respondents (mean 3.09). The results for age and education are statistically significant at p < 0.01.

#### 4.1.5 Trust in privacy

Results for T11 (Table 3) reveals that with regard to belief in the likelihood of privacy-related incidents, respondents tended to be neutral (mean 2.29). Levels of trust in privacy (likelihood) is lower for females, those aged 65+ and ISCED 5–6 respondents (mean 2.26, 2.25 and 2.25). Respondents aged 18–24 have slightly more trust in privacy (mean 2.32) as do ISCED 0–2 respondents (mean 2.31). The results for this variable are not statistically significant at p < 0.01.

Results for T12 (Table 3) shows that overall, respondents tended to disagree that the impact of privacy-related incidents is low (mean 3.33). This result is broadly consistent amongst all groups except for those age 18–24, who report feeling less negative about the impact of privacy-related incidents (mean 3.26); conversely, respondents aged 65+, and those with ISCED 0–2 education feel more negatively about the impact of privacy-related incidents. Results for this variable are not significant at p < 0.01.

Results for T13 (Table 3) shows that respondents tended to agree that the Facebook user data sharing controversy makes them less willing to own/use smart home devices (mean 2.44), with those aged 65+ feeling (mean 2.26) and ISCED 5–6 respondents (mean 2.42) even more discouraged. Respondents aged 35–49 (mean 2.55) and ISCED 0–2 respondents (mean 2.48) feel less discouraged than all other groups. The results for age and education are statistically significant at p < 0.01.

#### 4.1.6 Trust in security

Results for T14 (Table 3) shows that overall, respondents tended towards neither agreeing nor disagreeing that the likelihood of compromised security in smart home devices is high (mean 2.50). This result is consistent amongst all demographic groups. The results for this variable are not significant at p < 0.01.

Results for T15 (Table 3) shows that overall, respondents tended to be slightly negative about the impact of security-related incidents (mean 3.32), with those aged 65+ and ISCED 5–6 respondents feeling even more negative about trusting security of IoT (mean 3.36). On the other hand, respondents aged 18–24 and ISCED 3–4 respondents tended to be more neutral (mean 3.26 and 3.28), hence slightly less negative than average. The results for this variable are not statistically significant.

#### 4.1.7 Satisfaction, intention to use and intention to recommend

Finally, results for S16 (Table 3) shows that overall respondents felt only very slightly satisfied with their devices (mean 2.88). Male respondents (mean 2.94) were slightly more dissatisfied than women (mean 2.83). Respondents aged 18–24 and 35–49 were more satisfied (mean 2.47 and 2.75) than those aged 65+ (mean 3.20). ISCED 3–4 respondents were slightly more dissatisfied (mean 2.91) than respondents with ISCED 0–2 and ISCED 5–6 education (mean 2.86 and 2.85). The results for age are statistically significant at p < 0.01.

Results for I17 (Table 3) shows that, overall, respondents were marginally positive about their intention to use smart home devices in the future (mean 2.45), with respondents aged 18–24 being significantly more positive (mean 1.97) than average, and those aged 65+ (mean 2.84) or with ISCED 0–2 education less positive (mean 2.68). The results for age and education are statistically significant at p < 0.01.

Results for I18 (Table 3) shows that respondents were slightly positive about recommending smart home devices to their friends (mean 3.03), with males, lower educated respondents (mean 2.95, 2.93, and those aged 65+ (mean 2.74) being less likely to recommend smart home devices to others. In contrast, respondents aged 18–24 tended to be more positive about recommending IoT for the home to others (mean 3.33). The results for gender, age and education are statistically significant at p < 0.01.

### 4.2 Exploratory factor analysis

Having obtained a descriptive picture of attitudes toward smart home devices, we sought to develop an analytical model to assess key associations between the variables. Our questionnaire contained a total of 18 items, eight of which related to trust. In order to reduce this large number to a few core factors and study their impact on adoption, we used exploratory factor analysis (EFA). The purpose of EFA is to identify the factor structure of a set of variables to determine the number of factors and the pattern of factor loadings, i.e., the exact extent to which the variables influence these factors [79]. We decided to focus on obtaining factors from the eight trust-related variables (T8 = to T15, see Table 2) and used STATA to perform factor analysis.

In order to determine the minimum standard for proceeding with factor analysis, and the suitability of our data for structure detection, we ran a factor test in STATA, consisting of the Bartlett test of sphericity and the Kaiser-Meyer-Olkin (KMO) measure of sampling adequacy. The small p-value (0.00) obtained from Bartlett’s test of sphericity showed significance above the 95% level, indicating that there were sufficient intercorrelations to conduct factor analysis. Also, the rather high value obtained from KMO (0.83) indicated that factor analysis might usefully be applied to our data, since the closer the values are to 1.0, the more useful the analysis for the dataset.

We then ran principal component factor (PCF) analysis on STATA and obtained the results shown in Table 4. As shown in Table 4, Factor 1 explained about 48% of the total variance in the eight trust-related variables, the second factor explained 12%, the third 11% and the fourth 8%. Factors with an eigenvalue above 1 are usually retained. In our results, this applied only to Factor 1 (eigenvalue = 3.87), meaning that this factor explained the greatest proportion of the total variance.

**Table 4:** Results of principal component factor analysis (n = 2,101).

Factor	Eigenvalue	Proportion	Cumulative
Factor1	3.87	0.48	0.48
Factor2	0.98	0.12	0.61
Factor3	0.85	0.11	0.71
Factor4	0.62	0.08	0.79
Factor5	0.51	0.06	0.86
Factor6	0.46	0.06	0.91
Factor7	0.43	0.05	0.97
Factor8	0.27	0.03	1.00
chi2(28) = 6100.35		Prob>chi2 = 0.00	

We evaluated the strength of association between a given variable and the factor by looking at the commonality of each variable [79]. This was obtained by positing 1 minus the ‘uniqueness’ value of a variable (the variance unique to a specific variable and not shared with other variables). The greater the ‘uniqueness’, the lower the variable’s impact on the factor. For example, variable T14’s uniqueness value was 0.4, and hence its commonality was 0.6, showing that this variable was strongly related to the factor (see Table 5). The factor here accounted for 60% of variance in T14, dropping to 37% of variance in the T13 variable.

**Table 5:** Rotated factor loadings and unique variance of trust variables T8 to T15.

Variable	Factor1	Uniqueness
T8 Trust in competence	-0.69	0.52
T9 Trust in benevolence	0.67	0.55
T10 Trust in integrity	-0.66	0.56
T11 Likelihood of privacy breach	0.76	0.43
T12 Impact of privacy breach	-0.69	0.53
T13 Impact of controversy	0.61	0.63
T14 Likelihood of incident	0.78	0.40
T15 Impact of incident	-0.71	0.50

In order to be selective in how many and which variables to consider when describing the new factor, we focused on factor loadings, the correlation coefficients of the variable and the factor. To do so, we rotated the results using orthogonal varimax, and sorted them by factor loading (Table 5). Rotation maximises the interpretability of factor loadings but does not change other key statistics associated with it, such as the proportion of variance and commonality. This was a reason for focusing on factor loadings to facilitate factor interpretation [79]. Furthermore, the threshold value of a structure or pattern coefficient is 0.40 [79]. As Table 5 shows, the results obtained for factor loading were all above 0.40, indicating that all variables contributing to Factor 1 had a good factor loading.

Before looking at the characteristics of variables with the highest factor loadings, we calculated the internal consistency reliability or coefficient alpha of these results by running a Crohnbach’s alpha test in STATA. The internal consistency reliability coefficient (0.84) was above 0.80, indicating that there was good internal consistency reliability in this factor.

After this testing, we finally proceeded toward naming the new factor by examining the structure of variables with the highest factor loadings. We retained four variables with factor loadings either above or closest to 0.7 (T14 = likelihood of incident, T11 = likelihood of privacy breach, T15 = impact of incident and T8 = trust in competence; see Table 5).

### 4.3 Qualitative syntagmatic analysis

To frame qualitative analysis of the structure of the retained trust variables, we used syntagmatic analysis. This method, which is used for qualitative analysis of texts [80], draws on the notion of language as a superstructure, or *paradigm*, of substitutable elements or linguistic signs, which can then be combined in strings of signs, or *syntagms*. We therefore carried out a simple syntagmatic analysis of the structure of the variables by relating each key linguistic sign in a sentence to other linguistic signs occupying the same space in another sentence:

T14: I think the **likelihood** of the security of smart home devices being compromised and resulting in an *incident* (e.g. burglary, fraud) is high.
T11: I think the **likelihood** of the security of smart home devices being compromised and resulting in a *privacy/data breach* is high.
T15: I think the **impact** of the security of smart home devices being compromised and resulting in an *incident (e*.*g*. *burglary*, *fraud*) is low.
T8: I (would) fully trust smart home devices not to fail, and to function as I expect them to (competence).

The paradigms, or sets of two substitutable elements, retrieved through syntagmatic analysis were ‘likelihood, impact’ (in bold); ‘security incident, privacy/data breach’ (in italics); and ‘physical risk, performance risk’ (underlined). We then identified whether the paradigmatic element/features (‘likelihood’, ‘impact’ etc) were present in each of the question (see Table 6). For example, the structural features present in the variable T14 (= likelihood of incident) are ‘likelihood’, ‘security incident’ and ‘burglary or fraud’ (corresponding semantically rather than syntactically to physical risk). The structure of the sentence representing variable *T8* (= trust in competence) differed from that of the other variables, meaning that the feature-identification was made based on semantic links alone. For example, trusting a device not to fail refers to a concern about the likelihood of failure rather than its impact. It also relates primarily to performance risk (as defined by [69]) rather than physical risk.

**Table 6:** Scoring the presence of key paradigmatic elements against each sentence/variable.

Variable	Likelihood	Impact	*Privacy breach*	*Security incident*	Physical risk	Performance risk
T14 Likelihood of incident	1			1	1	
T11 Likelihood of privacy breach	1		1		1	
T15 Impact of incident		1		1	1	
T8 Trust in competence	1					1
**Total**	**3**	**1**	**1**	**2**	**3**	**1**

The results in Table 6 indicated that key aspects of the new trust factor obtained through factor analysis concerned primarily the *likelihood* of a *security-related incident* resulting in *physical risk*. Based on these prominent common features, we named the new trust factor ‘incident anxiety’ and turned it into a new trust ‘supervariable’ for incorporation into multivariate analysis to study its impact on adoption.

### 4.3 Ordered logistic multivariable regression

In the final part of the analysis, we focused on variables from our questionnaire that were applicable to the whole population (n = 2,101). We therefore excluded experience-related variables (E6 and E7) and the satisfaction variable (S16), which only applied to 1,422 respondents. We were selective in the variables for inclusion in our analysis because a statistical model with many variables has less statistical power [81]. We then set out to test the influence of the remaining independent variables–*awareness* (*A1 =* Awareness IoT, *A2 =* Awareness smart home), *ownership* (*O4 =* Ownership amount), *experience* (*E5 =* Experience length), *incident anxiety* (the new ‘supervariable’ obtained from factor analysis) and *recommendation* (*I18 =* Intention to recommend)–on the dependent variable (*I17* = Intention to use).

We opted to use logistic ordered multivariate regression to investigate the likelihood of technology adoption: multivariate because we wish to assess the relationship between the dependent variable and more than one independent variable [82]. logistic rather than linear because the dependent variable is categorical rather than continuous. Also, logistic regression allows analysis of multiple explanatory variables simultaneously, and their effect on the dependent variable, whilst also reducing the effect of confounding factors [81]. In other words, looking at multiple explanatory variables independently (through linear rather than logistic regression) would ignore covariance among the variables and the resulting associations would be subject to confounding effects. Finally, logistic regression is ordered because the dependent variable (*I17* = intention to use) presents ordered levels owing to the Likert scale on which it is based. The results obtained from our model through the ordered logistic multivariable regression are shown in Table 7.

**Table 7:** Results of Model 1 and Model 2 of ordered logistic regression including variables applicable to the whole population and the new ‘supervariable’ obtained from factor analysis, named ‘incident anxiety’ (N = 2,101).

	A. Model 1		B. Model 2	
	Ordered logistic regression showing log odds coefficient (*R*^2^ = 0.21)		Ordered logistic regression showing odds ratios (*R*^2^ = 0.21)	
Variables	Coefficient	95% CI	Odds Ratios	95% CI
**gender** 2 (male)	-0.10	(-0.28, 0.07)	0.90	(0.76, 1.08)
**Age**				
3 (25–34)	0.17	(-0.16, 0.50)	1.19	(0.85, 1.65)
4 (35–49)	0.52	(0.20, 0.83)***	1.67	(1.23, 2.29)***
5 (50–64)	0.89	(0.57, 1.21)***	2.44	(1.78, 3.36)***
6 (65+)	0.98	(0.66, 1.30)***	2.67	(1.94, 3.68)***
**Edu**				
2 (undergrad degree)	0.06	(-0.23, 0.36)	1.07	(0.79, 1.43)
3 (higher Diploma_	-0.03	(-0.38, 0.32)	0.97	(0.68, 1.38)
4 (A-Level)	0.16	(-0.13, 0.46)	1.18	(0.87, 1.59)
5 (GCSE)	0.12	(-0.20, 0.44)	1.13	(0.82, 1.55)
6 (primary education)	0.48	(-0.20, 1.16)	1.62	(0.82, 3.20)
7 (pre-primary education)	-0.02	(-1.34, 1.29)	0.98	(0.26, 3.67)
**A1** 2 (No)	0.33	(0.15, 0.50)***	1.39	(1.16, 1.65)***
**A2** 2 (No)	0.18	(-0.10, 0.46)	1.20	(0.90, 1.59)
**O4**	-0.20	(-0.26, -0.14)***	0.82	(0.77, 0.87)***
**E5**				
2 (0 to 1 years)	-1.37	(-1.62, -1.13)***	0.25	(0.20, 0.32)***
3 (2 years or more)	-1.72	(-1.97, -1.48)***	0.18	(0.14, 0.23)***
**Incident anxiety**	0.43	(0.23, 0.64)***	1.54	(1.26, 1.89)***
**I18**				
2 (somewhat agree)	-1.57	(-1.89, -1.25)***	0.21	(0.15, 0.29)***
3 (neither agree nor disagree)	-1.90	(-2.20, -1.61)***	0.15	(0.11, 0.20)***
4 (somewhat disagree)	-2.81	(-3.14, -2.48)***	0.06	(0.04, 0.08)***
5 (strongly disagree)	-3.60	(-4.01, -3.20)***	0.03	(0.02, 0.04)***

*R*^2^ is the goodness of fit

*P < 0.05

**P < 0.01

***P < 0.001

Variables’ levels are represented by dummy variables.

Since most of the independent variables are categorical (except for the ownership variable, *O4*), in the regression *they are represented by n-1 binary variables* (called dummy variables), where *n* indicates the initial number of levels of the variable [81]. Therefore, for example, the regression results for the education variable, *edu*, are presented only for values 2 to 7, rather than 1 to 7. The missing level is the ‘reference group’ and dummy variables are interpreted based on their distance/difference from this reference group. Given that the dependent variable, *I17* (= intention to use) has a categorical value itself, with values ranging from 1 = strongly agree to 5 = strongly disagree, positive or direct correlations will approach the highest value (the strongly disagree value), whilst negative correlations will approach the lowest value (strongly agree). In other words, positive correlations point to a decreased likelihood of intention to use, whereas negative correlations point to an increased likelihood. In the coefficients of Model 1 shown in the Table 7, it follows that only the results for age (except for its level 3), *A1 =* Awareness IoT, *E5 =* Experience length, ‘*incident anxiety’* and *I18 =* Intention to recommend, are statistically significant.

To better interpret these results, we followed Hoffman’s [83] guidelines for interpreting ordered logistic regression and decided to focus on odds ratios based solely on positive coefficients, rather than log-odds ratios based on both positive and negative coefficients (as in Table 7, Model 1). Therefore, we converted the logarithmic odds into odds ratios (Table 7, Model 2). The interpretation we produced is as follows:

*Age*: For a one-unit increase in age (excluding level 3 for which the results were not significant), the odds of the strongly disagree category versus the odds of the middle and low categories (neutral and strongly agree) for *i17 (=* Intention to use) increase by 1.6 times for people aged 35–49, 2.4 times for people aged 50–64, and 2.6 times for people older than 65, keeping all other variables in the model constant. In other words, from age 35, the older people are, the higher the odds that they will not want to use smart home devices in the future.

*A1* = Awareness IoT: The odds of strongly disagreeing with intending to use the technology in the future are 1.3 times higher amongst people who are *not* aware of the expression ‘IoT’ than people who *are* aware of it, keeping all other variables in the model constant. In other words, the less aware people are, the higher the odds (1.3 times) that they will not want to use the technology in the future.

*E5 =* Experience length: Keeping all other variables in the model constant, the odds of strongly disagreeing with intending to use smart home devices in the future amongst more IoT-experienced people (2+ years of experience) are low, at 0.1; these odds are slightly higher, at 0.2, for less IoT-experienced people (between 0 and 1 years of experience). In other words, the less experienced the users, the marginally higher the odds (0.2) that they will not want to use the technology in the future.

*04 =* Ownership amount: The odds of strongly disagreeing with intending to use the technology in the future amongst people who have no devices are 0.8 times the odds for people who do have devices, keeping all other variables in the model constant. In other words, the fewer smart home devices people own, the higher the odds (0.8 times) that they will not want to use the technology in the future.

*Incident anxiety*: For a one-unit increase in *incident anxiety*, the odds are 1.5 times higher for the strongly disagree category of *i17* versus the odds of the middle and low categories (neutral and strongly agree), keeping all other variables in the model constant. In other words, the higher the anxiety about the likelihood of a security-related incident resulting in physical risk, the significantly higher the odds (1.5 times) that people will not want to use it in the future.

*I18 =* Intention to recommend: For a one-unit increase in *i18* –from not wanting to recommend smart home devices to others (level 2) to wanting to do so (level 5)–the odds of strongly disagreeing about not wanting to use the technology in the future (*I17*) reduce from 0.2 to 0.02 times. In other words, the keener people are to recommend the technology to family and friends, the higher the odds (from 0.02 to 0.2 times) that they will want to use/keep using the technology in the future.

## 5. Discussion

The objective of this study was to develop an in-depth understanding of consumers’ responses and attitudes to smart home devices, using a nationally representative survey to measure levels of *awareness*, *ownership* and *experience of use (early adoption*, *smart functionality usage*, *habit) trust (general)*, *trust in privacy and security*, *satisfaction*, *intention* to use the technology in the future, and intention to *recommend* it to others. These variables were measured along with key socio-demographic factors that have been found to affect responses to technology, such as *gender* [84, 51, 85], *age* [58, 86, 52] and *education* [53, 87, 88]. Also, through factor analysis we studied the structure of the 8 trust variables to understand which ones were contributing the most to a single, stronger trust factor which we named ‘incident anxiety’. We then measured through an ordered logistic regression the likelihood that certain selected factors within *awareness*, *ownership*, *experience of use*, *‘incident anxiety’* and *recommendation* would impact on adoption through the *‘intention to use’* variable.

### 5.1 Awareness

Overall, significantly more people have heard of the expression ‘smart home’ than the expression ‘Internet of things’. Gender and education have an effect, such that males and more educated people tend to be more familiar with the expression ‘Internet of things’ than females and less educated people. The same applies to the expression ‘smart home’, but the difference is less pronounced. Furthermore, we found that the less aware people are about the expression ‘Internet of things’, the lower the odds of adopting the technology in the future. This is not surprising as the awareness of this specific expression is a sign of a wider technological awareness and disposition within the person. Awareness of the expression ‘Internet of things’ is a predicting factor for smart home adoption.

### 5.2 Ownership

Overall in the UK, people report owning almost 2 smart home devices each (1.86) but age and education affect *ownership*. The younger and the more educated people are, the more devices they report owning. This suggests that the oldest and least educated people are laggards in smart home device adoption. This trend is consistent with previous findings that older people find it more difficult to adopt new technologies [52] than younger people, and that, generally, adoption of new technologies is influenced by education (55; 8995]. We also found that the less smart home devices people own, the less likely they will want to adopt or continue using the technology in the future. The likelihood was not very high here but we can still interpret this result by assuming that owning devices is an indication of awareness in the technology or at least that a first experience with using them may have taken place—both these factors have been found to predict the likelihood of adoption (see discussion for ‘awareness’ and ‘experience of use’ below).

### 5.3 Experience of use

More people have adopted smart home devices two or more years ago than over the past year, suggesting the expansion of the consumer market for these devices has slowed down. According to our survey, a third of the UK population has not yet adopted smart home devices. Gender, age and education have an effect on the timing of adoption. Females have adopted smart home devices more than males over the past year and, currently, there are more inexperienced male users than females. This finding echoes traditional divisions in labour and roles [89] and so is unsurprising for a technology aimed at the domestic market. Given that women still tend to bear the burden of unpaid work relating to cooking, child care and housework more than men [90], females may hope that smart home devices will help them cope with domestic chores [91].

In terms of age, young people appear to be the earliest adopters of smart home devices, being the group that has used them for more than two years and has adopted the most over the past year. However, despite currently being laggards, older people seem initially to have constituted an early adoption group, surprisingly almost on *a par* with younger people. This may be because a significant proportion of older people have more disposable income and are less likely to be constrained by mortgage repayments and dependent children [92] and, hence, have more freedom to take risk of adopting the technology early on.

In terms of education, ISCED 3–4, followed by ISCED 5–6 were early adopters of smart homes and the group that have adopted the technology the most over the past year. Usually, the “more highly-educated individuals [who] tend to adopt innovations sooner than less-educated individuals” [93] but, over the past year, the laggards of ISCED 0–2 have been catching up. This is consistent with the fact that level of education does not affect long-term adoption of an innovation [88].

On average, UK consumers are neutral or only mildly in agreement with the statement that use of smart devices has become *routine*. However, this is affected by age, as older people are more likely to disagree. This may be because technology-related habit making for younger generations is more fluid, and with increased age, it is difficult to override a previous technology-related habit and adapt to change [58].

Although older people are early adopters in our survey, the fact that they report owning fewer smart home devices suggests that they are more discriminating in their choices, as evidenced by their interest in wireless webcams. This suggests that, far from responding uniformly negatively to technology, older people are selective in the technologies they use [52], rejecting some while embracing others.

Furthermore, we found that there is a slight likelihood that less experienced users will continue not wanting to use the technology in the future. The likelihood was only marginal here but we can still interpret this result by stating that without a first experience of device use, generating a pleasurable feeling, there is less incentive to kick-start a technological habit, or any habit in general.

Also, people generally report using only some of the smart functionalities of smart home devices, suggesting that the technology’s potential is not yet being fully exploited. This may translate into slower or even unreliable adoption. Significantly, age affects smart functionality usage, such that people over 65 years old report using even less functionalities than younger people.

### 5.4 Trust

When it comes to the *competence component of trust*, there is a tendency to disagree with the statement about trusting the reliability of smart home devices. In other words, people are unconvinced that smart home devices are reliable, although this may reflect a more general lack of trust in technology. Older people tend to be even less trusting than average about smart home devices’ reliability.

People tend to agree with the statement that unauthorised data collection (part of the benevolence component of trust) will influence their willingness to use smart home devices. This means that transparency about data collection is a concern and incidents involving poor data handling may potentially impact the adoption process. Age has an effect, as people aged 65 and over are less willing to use smart home devices in case of unauthorised data collection than younger people.

With regard to the integrity component of trust, people report feeling neutral about trusting businesses not to use data produced by smart home devices without their consent. This may suggest that consumers are adopting a ‘wait and see’ approach before forming an opinion. However, age and education are significant factors, as older and less educated people are less likely to trust the integrity of businesses providing smart home devices.

### 5.5 Trust in privacy and security

When it comes to trust in privacy, we measured attitudes in terms of both the likelihood and impact of privacy breaches. Overall, people tend to mildly agree that they are likely to risk privacy breach when using smart home devices. In other words, they are unconvinced that their privacy will not be at risk when they use smart home devices.

Also, when asked to evaluate the impact of a privacy breach, people tend to disagree that its impact will be low. This suggests that they expect the impact of a privacy breach to be fairly significant. This attitude is again affected by age, as those aged 65 and over tend to disagree even more that the impact will be low, whereas those aged 18–24 tend to disagree less. In other words, younger people tend to have less risk awareness of the impact of smart home-related privacy incidents than older people.

When it comes to trust in security, people tend to mildly agree that the risk of security breaches is likely when using smart home devices. No demographic factors seem be particularly significant in shaping this attitude. Also, there is a tendency to disagree that the impact of a security breach is low. Therefore, consumers expect that their security could be at risk and that security incidents would have a fairly significant impact.

IoT privacy and security risks have been widely reviewed in the literature [94, 95] and it is highly likely that these factors will impact negatively on the future adoption of these technologies [96]. It is difficult to envisage the IoT business community acting to address consumers’ privacy concerns without a strong regulatory push to incentivise this, yet some argue that the rapid pace of IoT development militates against effective policy interventions [97].

Businesses currently active in the smart home marketplace seem intent on avoiding making public statements about their position on privacy. This is understandable, given the evidence from our survey, which suggests that privacy is not yet a major issue for consumers. A notable exception is Apple, which has recently broken ranks and sought to distance itself from its competitors. Some observers believed that its statement ‘We at Apple believe that privacy is a fundamental human right’ [98] was designed to exploit Facebook’s discomfort following revelations about the personal data it had been harvesting from users’ mobile phones, rather than demonstrating a commitment to adopting privacy-preserving policies by minimising data collection and informed consent for the use of data. Apple is starting to make a push into the smart home devices marketplace but is not yet a big player. Therefore, it remains to be seen whether other companies will follow suit, and whether, for example, further revelations of data practices will succeed in making privacy into a major selling point for consumers. However, it is notable that a majority of respondents (54%) either strongly agreed or agreed that the Facebook data-sharing controversy had made them less willing to use smart home devices. In addition, a Pearson’s correlation test revealed that *Intention to use or continue to use smart home devices* (I17) is moderately negatively correlated with knowing that smart home devices allow companies or organisations to collect data about how users use them and hence about their domestic habits (Q9) (r = -.33). Hence, our survey provides evidence that consumers’ concerns about data privacy negatively impact adoption and that concern grows stronger with age.

With regard to security, there is little sign of businesses acting to address known and foreseeable vulnerabilities [99]. Furthermore, since security flaws in IoT devices already purchased and installed may be unfixable [100], many will remain vulnerable for the foreseeable future. Again, businesses seem intent on ignoring security when marketing their products. This position seems justified by the evidence from our survey, which suggests that this is not yet a major issue for consumers: while they profess awareness of the risks, these do not appear to have a strong influence on their intentions to buy. A Pearson’s correlation test revealed that intention to use or continue to use smart home devices (Q16) is moderately negatively correlated (r = -.25) with belief in the likelihood of a privacy/data breach (Q11); moderately negatively correlated (r = -.24) with belief about the magnitude of the impact of a privacy/data breach (Q15); as well as moderately negatively correlated (r = -.26) with belief in the likelihood of this resulting in an incident (e.g., burglary, fraud) (Q13). Hence, once again, our survey provides evidence that consumers’ concerns about security are likely to impact negatively on adoption.

Furthermore, we found that the most prominent factor affecting consumers trust is the anxiety about the likelihood of security-related incidents resulting in physical risk. The higher this anxiety, the significantly higher the odds that people will not want to adopt the technology or continue using it in the future. This result confirms that people are more concerned about personal safety than privacy-related issues as data integrity per se. It also suggests that people are more concerned about the likelihood that such events may happen rather than their impact, suggesting perhaps that people are not sufficiently educated as to what the risk and practical consequences of security-related incidents are.

### 5.6 Satisfaction

Overall, respondents reported feeling neutral about whether smart home devices had exceeded their expectations. This suggests that while devices may have met their basic expectations, consumers are still undecided about the benefits. Since indecision and doubt are the *loci* of change in habits [62], the possibility of failure is not yet excluded.

However, satisfaction is affected by age. Older people’s attitudes are more markedly on the negative side of neutral, while younger people tend to be more positive. There seems to be a minor education effect, with ISCED 3–4 people (the most experienced smart home device users) tending slightly more toward the negative side of neutral. A Pearson’s correlation test revealed that intention to use or continue to use smart home devices (Q16) is moderately negatively correlated (r = —.39) with trusting smart home devices not to fail and to function as expected (Q8).

### 5.7 Intention to use

Despite neutrality in satisfaction levels, overall, people tend to agree that they intend to continue to use smart home devices despite fairly low levels of trust; as noted above, this is negatively correlated with lack of trust (privacy, security) and with low expectations (reliability). This result points at contradictory consumer attitudes, where low trust, such as reflected in fear of security- and privacy-related incidents, conflict with positive attitudes reflected in future intention to use. A deeper qualitative understanding of attitudes subsumed in this contradictory finding might be beneficial for policymakers and businesses in steering adoption toward the positive spectrum of consumer attitudes.

There is a marked difference in terms of both age and education regarding intention to use smart home devices in the future, as 18–24 year olds agree with the statement, while those aged 65 and older tend to be neutral or negative side. Also, ISCED 0–2 people tend to be neutral or slightly negative, whereas ISCED 5–6 people tend to be neutral or slightly positive. This suggests that older and less educated people are the least interested in using smart home devices in the future, and that these might constitute market segments that will be lost to smart home adoption, unless their concerns are specifically addressed and targeted by policymakers and businesses.

### 5.8 Intention to recommend

People report feeling neutral about recommending smart home devices to others, possibly owing to uncertainty about their own levels of satisfaction and trust. However, age and education affect such attitudes: people aged 65 plus and those with ISCED 0–2 education report feeling less inclined to recommend devices. This suggests that, in time, the smart home–as previously with technology such as the Internet [101], mobile phones and social media [102]–may be characterised by a digital divide that will exclude older and less educated members of the population. We also found that there is a weak relation between intending to recommend smart home devices to family and friends, and wanting to use/keep using the technology oneself. This result shows again uncertainty in regard to adoption.

## 6. Conclusions

### 6.1 Summary

Our survey aimed to reveal the influence of factors on consumers’ meanings and trust in the smart home. Innovation processes are typically complex, and our results can provide only a snapshot of these factors. Their influence may change over time, and other factors may yet emerge. Adoption constructs allowed us to measure the awareness and current penetration of smart home devices amongst UK consumers, and to explore demographic factors. Overall, with regard to the extent to which smart home devices have been adopted in the UK, we can conclude that, despite a fairly significant take-up (two thirds of UK consumers own smart devices) and the more pronounced enthusiasm of specific demographics (younger and more educated people), smart home device adoption at the time of data collection was still at the ‘early majority’ stage. Exploring acceptability allowed us to understand how the public interprets and hence perceives the smart home, particularly with regard to trust, satisfaction with and likelihood of using them in the future and recommending them to others.

For a more in-depth analysis, we broke down the responses according to gender, age and education. Overall, our research reveals that age is the most influential factor influencing smart home devices’ adoption and acceptability. More specifically, gender may have initially affected the adoption of smart home devices, with males currently showing slightly more favourable attitudes than females. Furthermore, age is the most decisive factor, in that younger people are more likely to hold favourable attitudes toward smart home devices and more likely to be wanting to use it in the future. However, older people have also been found to be early adopters at almost the same level as younger people (but note that older people are also less satisfied).

Overall, we conclude that the following acceptability factors frame consumers’ meanings and trust in the smart home and undermine the adoption of smart home devices in the eyes of UK consumers:

- Fairly low levels of trust in IoT, particularly regarding the likelihood of incidents resulting in physical risk
- Fairly low levels of satisfaction
- Younger respondents’ low risk awareness
- Older respondents’ resistance to IoT
- Less well-educated respondents’ resistance to IoT

### 6.2 Theoretical and practical implications for businesses and public policy

From a SCODT perspective, our findings suggest that the meaning and value proposition of the smart home is subject to conflicting interpretations for consumers. Businesses are still actively promoting visions of what the smart home means for consumers (e.g., convenience, economy, home security) through advertising campaigns aimed at ‘configuring the user’ [29] or, perhaps more accurately, ‘configuring the product’ (see below). In other words, businesses are trying to influence consumers to focus only on specific contextual factors that will uniquely frame a positive interpretation, rather than addressing concerns about risk. However, at the same time, as we see from our survey results, consumers are actively comparing their interactional experiences against these visions and are coming up with different interpretations and meanings from those that business is trying to promote. Hence, the capacity of businesses to control the narrative over their products is unclear. As networked individuals, consumers are able to engage in social learning by sharing their experiences on social media [18] about, for example, the privacy and security risks of smart home devices. Such concerns are amplified by regular media reports of security breaches, such as hackers gaining access to data or gaining control of smart home devices and alleged privacy violations. The smart home–and IoT more generally–represents the leading edge of the generation of digital innovations that emerged in the early 2000s. Their distinguishing characteristic is hyper-connectivity and for the public their potential will have first become manifest through social media platforms such as Facebook and Twitter. In the beginning, these were celebrated as liberating, but subsequent events, such as the rise of cyber hate and the Facebook-Cambridge Analytica scandal, have presented the public with a darker, more troubling reality. Indeed, recent academic critiques characterise the underlying economic model of the new wave of digital innovations as ‘surveillance capitalism’ [103], which is distinguished by the harvesting of data and its analysis for the commodification of human activity. From this perspective, *‘you are the product’*, *regardless of whether you pay for the service*. The phrase ‘if it’s free you are the product’ has become synonymous with services such as Facebook, Google and Twitter. With IoT, people pay but arguably are still the product. Hence, the meaning of the smart home is at risk of becoming strongly polarised: closure over its meaning–and for IoT more generally–may be a long way off and what form it will take remains an open question [104]. For the time being, our results highlighted the prominence of ‘incident anxiety’ as one of the key interpretational constraints [105] specifically a psychological one, that frame consumers’ meanings in the smart home. Therefore, in our study, *anxiety about likelihood of security-incident emerges as a prominent sociotechnical affordance*, *and influence on consumers’ attitudes*.

Our study flags up clear challenges to smart home adoption in the UK for both businesses and policymakers. For example, our findings make a case against the purely economically-driven approach to the adoption of IoT on which recent IoT literature has tended to rely, focusing on traditional acceptance models untailored to the uniqueness of IoT and ignorant of various facets of IoT trust. Such an approach would be counter-productive, because businesses would have difficulty reaching potentially important markets. Excluded or poorly represented consumer segments would have no opportunity to influence development at a sufficiently early stage. In the case of the smart home, this would suggest that the well-being of an ever-increasing proportion of society (considering the ageing population in Western economies) will be neglected, increasing the risk that society may become more polarised between the ‘digitally included’ and the ‘digitally left behind’. This gap may become harder and harder to bridge. In turn, this may have detrimental effects on health, sustainability and the economy. The business sector and policymakers need to work closely together to address barriers to adoption and acceptability of the smart home now and in the future.

In particular, the focus should be on 1) countering consumers’ lack of trust through, for example, committing to adopting approved standards for security, and providing greater clarity about how consumers’ personal data are being used; furthermore, the emergence of ‘incident anxiety’ as a risk that smart home technology affords, points to the need to improve the design of devices in regard specifically with their reliability, to decrease the risk likelihood and increase trust in consumers. Also, as people are currently mainly focussed on risk-likelihood and may underestimate risk-impact, there emerges the need for education on the impact of security-related incidents, to give consumers the resources to indeed deal with such incidents and make them more physically and emotionally resilient. Higher level of incident-resilience in the population may eventually result in higher level of trust in the devices and consequently, a wider, healthier and more sustainable market for smart home technology; and 2) working with organisations representing consumers to developing business models and value propositions that resonate with the needs, expectations and concerns of a broad base of consumers.

To achieve these objectives, it is vital that businesses providing smart home devices and services make sustained efforts to understand how interpretations, attitudes and patterns of use change, and what their implications might be for evolving products and refining the value proposition. The goal should be to track the innovation trajectory over time and at scale, and thus provide evidence for device and service evolution. Of course, these businesses already have data on how consumers use smart home devices to enable them to do this. Quantitative studies at scale should be complemented with smaller, case study-based qualitative investigations to shed light on the reasons underlying changes in users’ attitudes and behaviour.

### 6.3 Limitations of the study and avenues for future research

Concerning sample, the online panel-based sample recruitment used in our survey offered the benefit of assembling a carefully composed, nationally representative sample efficiently and quickly. However, our sample is less likely to include the computer, Internet and smart phone illiterate, because respondents who are confident enough to sign up to an online panel, may also be more confident around new Internet-based technology than those who are not. Also, our research captured social divides reflected in demographic factors such as gender, age and education, but future research might complete this picture by including other demographic factors, such as ethnicity, geographical location, income and even health, since the safety of people living with chronic disabilities or illnesses could be supported by IoT solutions for the home, and perhaps make the technology feel more useful, desirable and acceptable. In terms of methods, the single, strong factor we identified through factor analysis, in order to best describe trust, might be tested in a new statistical model of technology acceptance tailored to the IoT. Future research might also look at performing further factor analysis on a longitudinal basis, to see if the structure of the trust factor changes across time, perhaps switching from anxiety of incident-likelihood to anxiety of incident-impact, or from security-related incident anxiety to data-privacy breach anxiety. These changes may happen following any policy, regulation and educational intervention as suggested in our discussion above, or any notable future incidents or privacy breaches involving the smart home technology. Knowing the ways in which changes in meanings and trust in the smart home may imply changes in smart home adoption pattern–a fact that both businesses and policy makers may want to keep track of to ensure a fairer, healthier, more prosperous and more sustainable society.
